# Anti-Mycotoxin Agents in Pig Production Through the Lens of Circular Economy and Life Cycle Assessment: A Comprehensive Review

**DOI:** 10.3390/toxins18080325

**Published:** 2026-07-27

**Authors:** Georgios I. Papakonstantinou, Christos Eliopoulos, Dimitrios Arapoglou, Nikolaos Tsekouras, Katerina Manolakou, Labrini V. Athanasiou, Dimitrios Gougoulis, Soultanidis Aris, Vasileios G. Papatsiros

**Affiliations:** 1 Clinic of Medicine, Faculty of Veterinary Medicine, University of Thessaly, 43100 Karditsa, Greece; nitsekou@vet.uth.gr (N.T.); lathan@vet.uth.gr (L.V.A.); dgoug@uth.gr (D.G.); stasiaris@gmail.com (S.A.); 2 Institute of Technology of Agricultural Products, Hellenic Agricultural Organization-Demeter (HAO-Demeter), 14123 Athens, Greece; christoseliopoulos@hotmail.com (C.E.); dimarap@yahoo.com (D.A.); 3 Department of Animal Husbandry and Nutrition, Faculty of Veterinary Medicine, University of Thessaly, 43100 Karditsa, Greece; kmanol@vet.uth.gr

**Keywords:** mycotoxins, pigs, anti-mycotoxin agents, multicomponent detoxifier, circular economy, life cycle assessment, sustainability, oxidative stress

## Abstract

Mycotoxin contamination of cereal-based pig feeds is a persistent threat to swine health and global pork safety. Pigs are uniquely susceptible to mycotoxicosis, and the principal toxins—aflatoxins (AFs), deoxynivalenol (DON), zearalenone (ZEN), fumonisins (FUMs), and ochratoxin A (OTA)—cause impaired growth, reproductive failure, hepato- and nephrotoxicity, immunosuppression, and carry-over residues in edible tissue. Anti-mycotoxin feed additives, including mineral adsorbents, biotransforming agents, and multicomponent mycotoxin-detoxifying agents (MMDAs) combining clays, phytogenic antioxidants (curcumin, silymarin), and postbiotics (yeast cell wall, hydrolyzed yeast), constitute the primary mitigation strategy. Beyond animal health, these agents play underappreciated roles in both the circular economy (CE)—enabling safe valorization of by-product feed ingredients and reducing feed waste—and in the life cycle assessment (LCA) sustainability profile of pork production, where feed conversion ratio (FCR) drives 22–95% of greenhouse gas emissions. Field-based in vivo studies in sows and weaned piglets, integrating plasma oxidative stress biomarkers (TBARS, protein carbonyls, total antioxidant capacity) with performance and reproductive endpoints, provide the most comprehensive real-world evidence for MMDA efficacy to date. This review synthesizes mycotoxin toxicology, anti-mycotoxin agent mechanisms, clinical evidence, and the CE/LCA sustainability framework, proposing integrated mycotoxin management as a One Health imperative in modern swine production.

## 1. Introduction

Mycotoxin contamination of cereal grains and compound feeds is a near-universal agricultural challenge of profound economic, veterinary, and environmental significance. More than 400 structurally diverse mycotoxins, produced principally by *Aspergillus* spp., *Fusarium* spp., and *Penicillium* spp., contaminate feed at pre- and post-harvest stages under the influence of climate, agricultural practices, and storage conditions [1]. Global surveillance consistently shows that 60–80% of feed samples harbor at least one mycotoxin, and multi-mycotoxin co-contamination predominates in commercial pig production [2,3].

Swine are among the most mycotoxin-susceptible livestock owing to cereal-heavy diets and a monogastric gastrointestinal tract with limited microbial biotransformation capacity. Consequences encompass reduced average daily gain (ADG), impaired feed conversion ratio (FCR), intestinal barrier dysfunction, hepato- and nephrotoxicity, reproductive failure, and immunosuppression predisposing to secondary infections [4,5]. Economic losses attributable to the three principal mycotoxins (AFs, DON, FUMs) were estimated at USD 1.4 billion annually in the United States alone, excluding downstream human health costs [2].

Feed production is the dominant environmental hotspot in pig production: life cycle assessment (LCA) studies assign 22–95% (mean 59 ± 18%) of total greenhouse gas (GHG) emissions to feed, with FCR as the single most predictive variable across all major LCA impact categories (correlation typically >0.95) [6,7]. Mycotoxin-induced FCR deterioration directly worsens the environmental footprint of pork production across global warming potential (GWP), eutrophication potential (EP), acidification potential (AP), and land occupation (LO). Conversely, antimycotoxin agents that restore FCR generate proportional co-reductions across all LCA categories.

**Figure 1:**
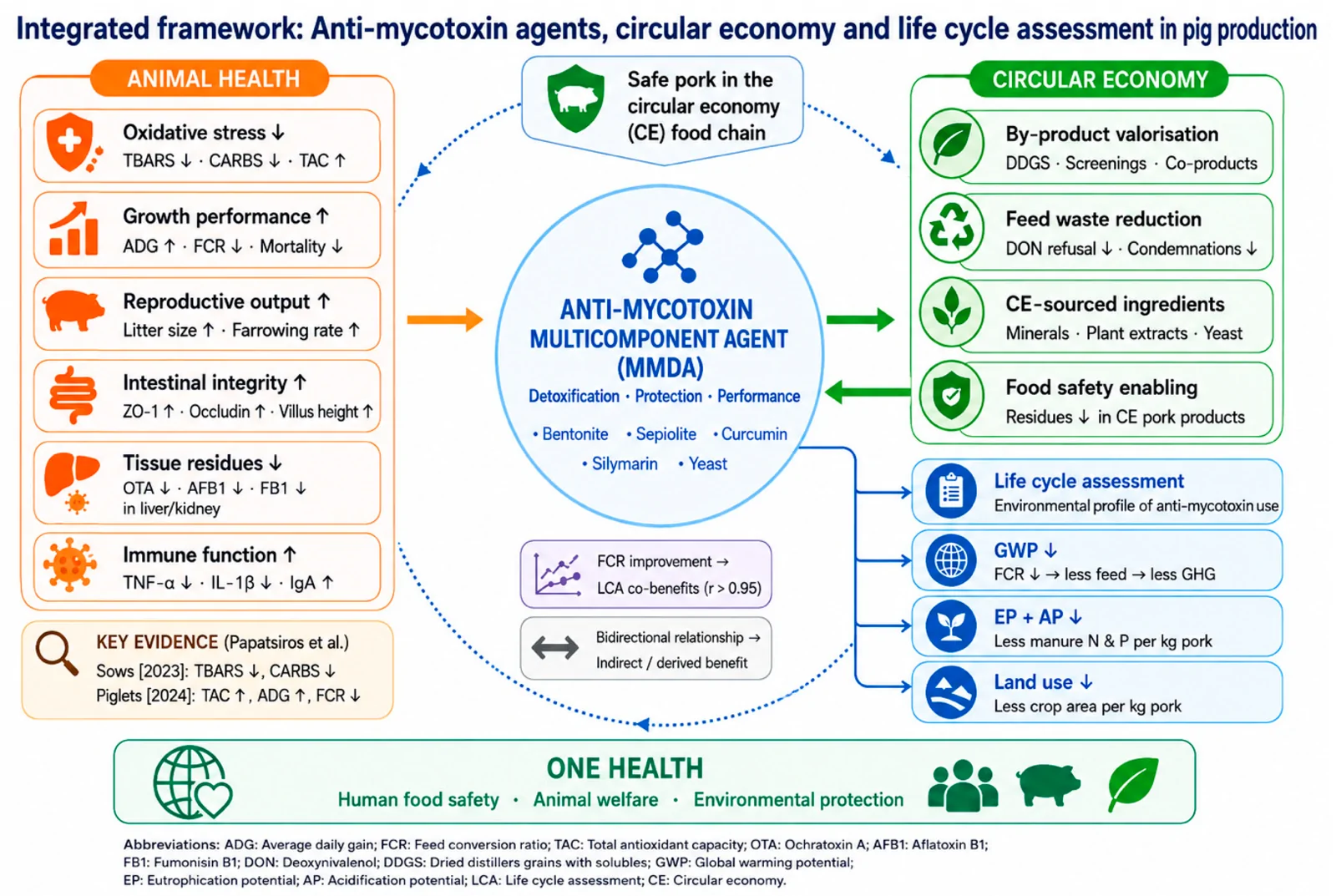
Integrated conceptual framework linking multicomponent mycotoxin-detoxifying agents (MMDAs) in pig production to circular economy (CE) and life cycle assessment (LCA) objectives. The MMDA (purple, center) connects to three outer domains: animal health (coral, left) showing six clinical benefit categories; circular economy (green, top-right) showing four valorization and waste-reduction benefits; and LCA (blue, bottom-right) showing three environmental co-benefits. The One Health banner (teal, base) underpins all domains. The key evidence badge (amber) highlights the field-based biomarker outcomes from Papatsiros et al. [10,11]. Solid bidirectional arrows indicate direct relationships; dashed arrows indicate indirect or derived benefits. GWP, global warming potential; EP, eutrophication potential; AP, acidification potential; TBARS, thiobarbituric acid reactive substances; CARBS, protein carbonyls; TAC, total antioxidant capacity; ADG, average daily gain; FCR, feed conversion ratio.

Circular economy (CE) principles further position antimycotoxin agents as enabling technologies: by facilitating the safe use of mycotoxin-contaminated cereal fractions, food-industry by-products, and former foodstuffs in pig diets, they keep valuable biomass in productive use and mitigate the food safety risks inherent in circular feed formulations [8,9]. Figure 1 presents the integrated conceptual framework connecting these dimensions.

This review integrates veterinary evidence on anti-mycotoxin agent efficacy in pigs—including the field-based MMDA studies by Papatsiros et al. [10,11,12]—with CE and LCA sustainability frameworks, proposing mycotoxin management as a One Health imperative in modern swine production.

## 2. Principal Mycotoxins in Swine: Occurrence, Toxicity, and Sustainability Costs

### 2.1. Global Occurrence and Climate Change

The global contamination landscape in pig-relevant cereals is dominated by Fusarium toxins (DON, ZEN, FUMs, T-2/HT-2) in temperate regions and aflatoxins in tropical and subtropical zones. Surveillance of Serbian maize (2021–2023) detected AFB1 above the human consumption limit (5 µg/kg) in 73.2% of samples in the drought year 2022, with fumonisins the most prevalent contaminant and frequent co-contamination documented [13]. Surveys of complete feeds in Southern Romania (2021–2024) confirm sub-chronic multi-mycotoxin co-exposure as the dominant commercial pig exposure pattern [14]. Climate change is projected to expand Aspergillus ecological range into temperate European regions, amplifying the mycotoxin management burden in CE-consistent feed systems that incorporate co-products.

Table 1 summarizes reported mycotoxin occurrence and co-contamination in swine-relevant cereals and complete feeds across recent European and global surveys, underscoring the predominance of multi-mycotoxin co-exposure in commercial pig production.

### 2.2. Mechanisms of Porcine Mycotoxicosis

AFB1 undergoes hepatic CYP450 bioactivation to the reactive AFB1-8,9-epoxide, which forms mutagenic DNA adducts, causing hepatocellular necrosis, cholestasis, and liver enzyme elevation. Immunosuppression is documented below the EU guidance threshold (10 µg/kg) [15]. A field study by Papatsiros et al. [12] using LC-MS/MS liver analysis at slaughter across commercial Greek finisher farms detected FB1 at 2.91–8.30 µg/kg alongside OTA at 0.54 µg/kg, with histopathological hepatocellular lesions confirming subclinical exposure in the absence of overt clinical signs.

DON binds the 60S ribosomal subunit, triggering the ribotoxic stress response with downstream MAPK activation, pro-inflammatory cytokine upregulation (TNF-α, IL-1β, IL-6), and apoptosis in intestinal epithelial cells. Loss of tight junction proteins (ZO-1, occludin, claudin-1) increases paracellular permeability [16,17]. DON crosses the placental barrier and is secreted in sow colostrum and milk [18]. ZEN is a phenolic resorcyclic acid lactone that preferentially binds estrogen receptor-α; its principal porcine metabolite α-zearalenol (α-ZEL) displays 3–4-fold greater estrogenic potency [19]. Reproductive toxicity includes vulvovaginitis, uterine enlargement, and embryonic resorption at dietary concentrations as low as 1 mg/kg [20]. FUMs (FB1, FB2) inhibit ceramide synthase, disrupting sphingolipid metabolism and causing hepatic lesions and immunosuppression chronically [21]. FB1 induces oxidative stress in pig vascular endothelium, elevating MDA and reducing antioxidant enzymes [22]. OTA bioaccumulates in pigs (plasma half-life ~88.8 h) [23], targeting the renal proximal tubule and generating mycotoxic porcine nephropathy with carry-over to pork products [24].

The toxidynamic pathways at the molecular level culminate in a small number of cellular targets that account for the multi-organ involvement seen in porcine mycotoxicosis. The major activating enzymes of AFB1 in the liver are the cytochrome P450s CYP1A2 and CYP3A, which form the exo-8,9-epoxide reactive metabolite that then reacts with guanine at the N7 position to produce the pro-mutagenic compound AFB1-N7-guanine adduct, and subsequently causes depurination-induced G → T transversion at codon 249 of the tumor suppressor gene TP53; the major mechanism of AFB1 detoxification is conjugation by glutathione S-transferase, whose reduced hepatic activity contributes to the increased species sensitivity of the pig to this mycotoxin [2,25]. AFB1 binds the A site of the 28S rRNA of the 60S ribosomal subunit and induces the ribotoxic stress response via double-stranded RNA-activated protein kinase (PKR) and hematopoietic cell kinase (Hck), leading to activation of p38, ERK and JNK mitogen-activated protein kinases, transcription through the NF-κB pathway of TNF-α, IL-1β and IL-6, and caspase-dependent cell death of enterocytes and immune cells. Feed rejection and vomiting responses are peripheral and occur via enteroendocrine release of cholecystokinin, glucagon-like peptide-1 and 5-hydroxytryptamine on vagal afferent pathways [17,21]. Zearalenone (ZEN) and its reductive metabolites α- and β-zearalenol, produced by 3α/3β hydroxysteroid dehydrogenases, have high binding affinities to estrogen receptors α and β, resulting in hyperestrogenism, granulosa cell proliferation and gonadotrophin feedback disruption [19]. Fumonisins are competitive inhibitors of ceramide synthase (sphinganine N-acyltransferase), resulting in an increased free sphinganine/sphingosine ratio and disruption of sphingolipid-related membrane and signaling functions, with ensuing oxidative stress and apoptosis in hepatocytes and vascular endothelial cells [21,22]. Ochratoxin A (OTA) shows a dual mechanism with inhibitory effects on phenylalanyl tRNA synthetase (which stops protein synthesis), mitochondrial dysfunction, lipoperoxidation and oxidative DNA damage in the renal proximal tubule [23,24].

### 2.3. Economic and Sustainability Dimensions

Mycotoxin-induced FCR deterioration imposes a computable environmental cost: in LCA frameworks where FCR correlates >0.95 with GWP, EP, AP, and LO [6,7], a 10% FCR deterioration from mycotoxicosis translates to a proportional increase across the entire environmental impact profile of pork production. Sow reproductive failures attributable to ZEN or DON-induced immunosuppression inflate the whole-enterprise FCR (FCRwe)—the parameter driving the full pork LCA burden per kg live weight. Table 2 summarizes the principal mycotoxins, EU thresholds, toxic targets, and sustainability implications.

## 3. Classification and Mechanisms of Anti-Mycotoxin Agents

**Figure 2:**
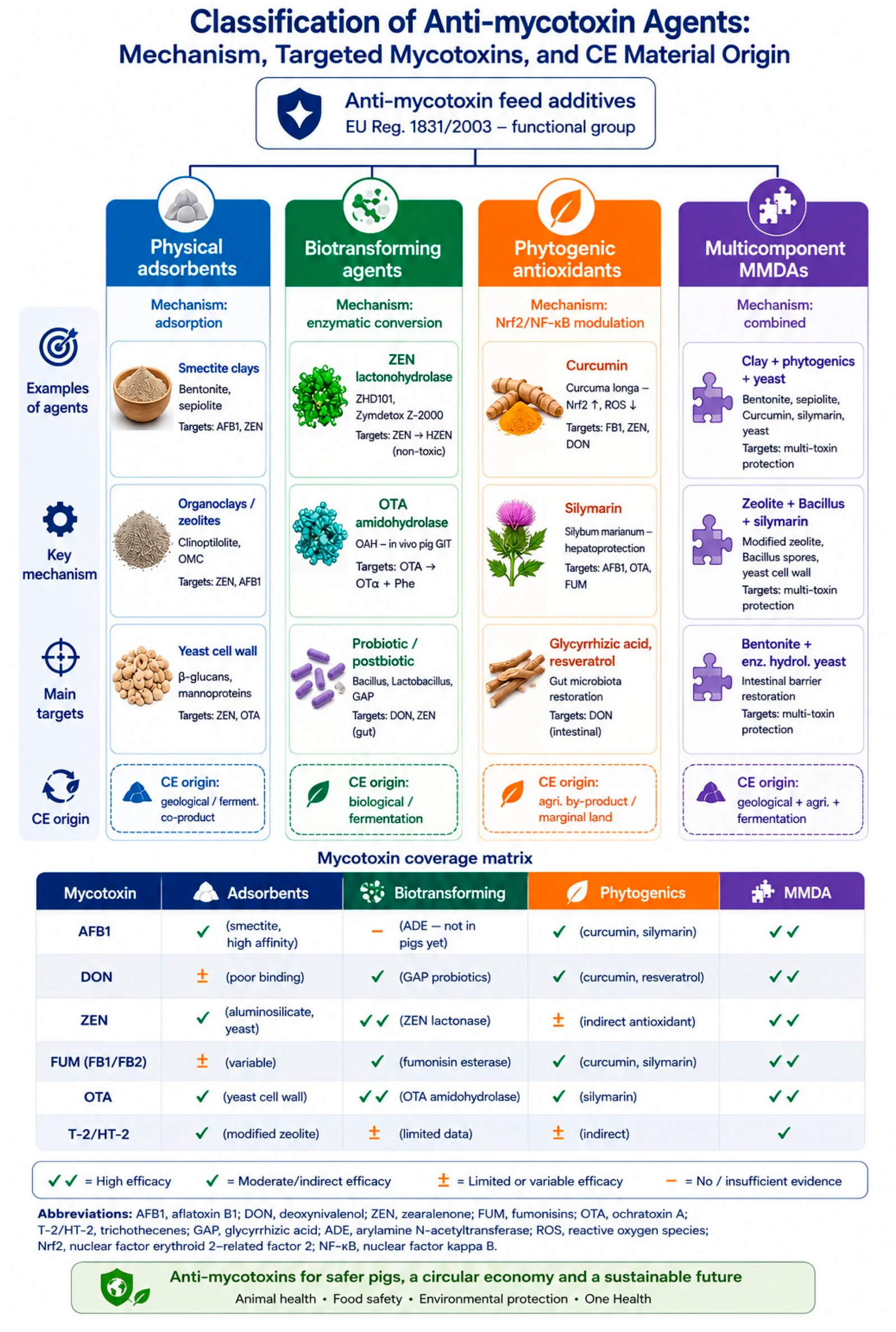
Classification of anti-mycotoxin agents in pig production by mechanism of action, key active components, targeted mycotoxins, and circular economy (CE) material origin. Four main branches are shown: (1) physical adsorbents (gray)—smectite clays (bentonite, sepiolite) [26,29], zeolites/organoclays [26], and yeast cell wall β-glucans [26,30]; (2) biotransforming agents (teal)—ZEN lactonohydrolase [27,31], OTA amidohydrolase [28], and probiotic/postbiotic cultures [32,33]; (3) phytogenic antioxidants (amber)—curcumin [34,35,36], silymarin [34], glycyrrhizic acid/resveratrol [32,37]; (4) multicomponent MMDAs (purple)—three representative formulations with key citations [10,11,30,38,39]. Dashed CE-origin badges indicate material type: gray = geological, teal = biological/fermentation, amber = agricultural by-product, purple = composite CE sources. The coverage matrix (bottom) summarizes relative efficacy for six mycotoxins across all four agent classes. ✓✓ high efficacy; ✓ moderate/indirect; ± limited or variable. AFB1, aflatoxin B1; DON, deoxynivalenol; ZEN, zearalenone; FUM, fumonisins; OTA, ochratoxin A; HZEN, hydrolyzed zearalenone; MMDA, multicomponent mycotoxin-detoxifying agent.

Figure 2 presents the classification of anti-mycotoxin agents by mechanism of action, sub-components, targeted mycotoxins, circular economy material origin, and mycotoxin coverage efficacy matrix.

### 3.1. Physical Adsorbents

Adsorbing agents bind mycotoxins within the gastrointestinal lumen, forming non-absorbable complexes excreted in feces. Aluminosilicate clays (montmorillonite, bentonite, smectite) adsorb planar polar AFB1 via electron donor–acceptor interlayer interactions with >92% in vitro efficiency across all products in porcine GI simulation models, while achieving <20% DON binding [27]. Sepiolite’s fibrous tunneled structure provides complementary adsorption geometry. Organically modified clays extend affinity to less-polar ZEN. Yeast cell wall β-1,3/1,6-glucans and mannoproteins adsorb ZEN and OTA through hydrophobic interactions while additionally modulating immunity [28]. From a CE perspective, mineral adsorbents derive from naturally occurring geological resources, and yeast cell wall fractions are recovered from brewing and bioethanol fermentation co-product streams [28].

### 3.2. Biotransforming and Biodegrading Agents

Biotransforming agents catalyze mycotoxin conversion into non- or less-toxic metabolites. Zearalenone lactonohydrolase (e.g., Zymdetox Z-2000 from Bacillus subtilis) cleaves the ZEN lactone ring, yielding hydrolyzed ZEN (HZEN) and decarboxylated HZEN with negligible estrogenicity; a controlled gilt trial confirmed effective mitigation of ZEN-induced reproductive toxicity [32]. OTA amidohydrolase (OAH) hydrolyzes OTA to non-toxic ochratoxin α and phenylalanine within the pig GI tract [33]. Glycyrrhizic acid combined with compound probiotics (GAP) in DON-challenged weaned piglets recovered intestinal gene expression profiles, reversed microbiota dysbiosis, and ameliorated growth performance [40].

The reaction pathways involved in the transformation of major mycotoxin-degrading enzymes have been described in detail. The zearalenone lactonohydrolase enzyme cleaves the ester linkage of the lactone ring of ZEN to form non-estrogenic hydrolyzed ZEN, which then spontaneously decarboxylates to decarboxylated, hydrolyzed ZEN, as ring opening destroys the geometry required for estrogen-receptor binding, thereby eliminating any estrogenic activity [32,41]. Ochratoxin A amidohydrolase hydrolyses the amide bond between L-β-phenylalanine and the isocoumarin ring of OTA, forming the non-toxic compounds ochratoxin α and phenylalanine [33]. Hydrolysis of the fumonisin carboxyl esters (by enzymes such as FumD) sequentially removes the two tricarballylic acid side chains to form hydrolyzed FB1, which is then further modified by a fumonisin aminotransferase to produce hydrolyzed FB1 lacking the ability to inhibit ceramide synthase; this compound is the basis of the authorized fumonisin esterase [34,41]. The two microbial detoxification pathways of deoxynivalenol involve oxidation–reduction in C3 by a DON epimerase system, converting DON to the much less toxic 3-epi-DON, and anaerobic de-epoxidation of the 12,13-epoxide to de-epoxy-DON (DOM-1) [34]. Degradation of aflatoxin B1 by micro-organisms occurs through oxidoreductase-mediated opening of the furan ring and lactone structure, thus reducing its mutagenicity [37]. Because each enzyme is highly substrate-specific, enzymatic biotransformation is deployed within multicomponent formulations alongside broad-spectrum adsorbents to achieve coverage of co-occurring mycotoxins.

### 3.3. Phytogenic Antioxidant Agents

Curcumin (*Curcuma longa*) neutralizes ROS via direct radical scavenging and Nrf2/ARE pathway activation, inducing SOD, CAT, and GSH-Px and downregulating TNF-α, IL-1β, and IL-6. Silymarin (*Silybum marianum*) stabilizes hepatocyte membranes, stimulates ribosomal RNA synthesis and hepatocyte regeneration, and provides complementary radical scavenging activity. In vitro efficacy against OTA, FB1, ZEN, and DON hepatotoxicity is established for both of them [35]. Phytobiotics combined with mycotoxin adsorbents outperformed adsorbent alone in mitigating multi-mycotoxin AF + FUM challenge effects in growing pigs [36].

Beyond curcumin and silymarin, a broader repertoire of phytogenic antioxidants has been evaluated as anti-mycotoxin agents in monogastric species. Grape-derived proanthocyanidins and resveratrol, green-tea catechins (notably epigallocatechin-3-gallate), quercetin, olive-pomace polyphenols, glycyrrhizic acid from liquorice, and the microalga *Spirulina platensis* act through combinations of direct radical scavenging, Nrf2/ARE-mediated induction of endogenous antioxidant enzymes, chelation of pro-oxidant metal ions, inhibition of CYP450-dependent toxin bioactivation, and suppression of NF-κB-driven inflammation [30,35,38]. In poultry and pig models, curcuminoids from *Curcuma longa* and grape-seed proanthocyanidins have reduced AFB1-induced oxidative damage and tissue residues, while *Spirulina*- and turmeric-based nutraceuticals improved redox status and performance under mycotoxin or oxidative challenge [30,38]. These agents are particularly relevant to circular feed systems because most are recovered from agro-industrial by-product streams (grape pomace, olive pomace, citrus peel, spent turmeric rhizome), aligning their sourcing with CE principles. Table 3 summarizes the principal phytogenic anti-mycotoxin agents, their botanical source, main active principles, and predominant protective mechanisms.

### 3.4. Multicomponent Mycotoxin-Detoxifying Agents (MMDAs)

MMDAs combine mineral adsorbents, biotransforming components, and phytogenic/postbiotic agents in a single formulation, addressing the limitations of single-mode agents under multi-mycotoxin contamination. The convergence of multiple mechanisms—physical binding of polar toxins, enzymatic biotransformation, antioxidant protection against residual toxin-induced cellular damage, and immunomodulatory support—mirrors the systems-thinking of CE approaches.

## 4. Clinical Evidence for MMDA Efficacy in Pigs

### 4.1. Evidence in Weaned Piglets

In a landmark field study, Papatsiros et al. [11] evaluated the MMDA (bentonite, sepiolite, curcumin, silymarin, yeast cell wall, hydrolyzed yeast) in 150 weaned piglets per arm across two independent commercial Greek farms, from day 28 to day 70. The standardized oxidative stress biomarker triad—TBARS (µmol/L), protein carbonyls (CARBS, nmol/L), and total antioxidant capacity (TAC, mmol/L)—was measured in plasma at days 45 and 70, with histopathological liver examination at day 70. The MMDA group showed statistically significant (*p* < 0.05) reductions in TBARS and CARBS and increases in TAC at both timepoints on both farms, alongside significantly improved ADG, FCR, and reduced mortality [11]. The FCR improvements documented under naturally contaminated field conditions provide the real-world performance recovery parameters required to populate the LCA environmental benefit calculation described in Section 3.2.

Supporting controlled trial evidence, Deng et al. [31] demonstrated that bentonite combined with enzymatically hydrolyzed yeast improved the villus height: crypt depth ratio and immune response in newly weaned pigs under combined FUM and AF challenge. Raj et al. [29] showed that a zeolite–Bacillus–yeast–silymarin MMDA at 1.5–3.0 g/kg in 168 weaned pigs challenged with DON (1.2–1.5 mg/kg) and ZEN (0.9 mg/kg) improved ADG and FCR and significantly reduced DON kidney residues.

**Figure 3:**
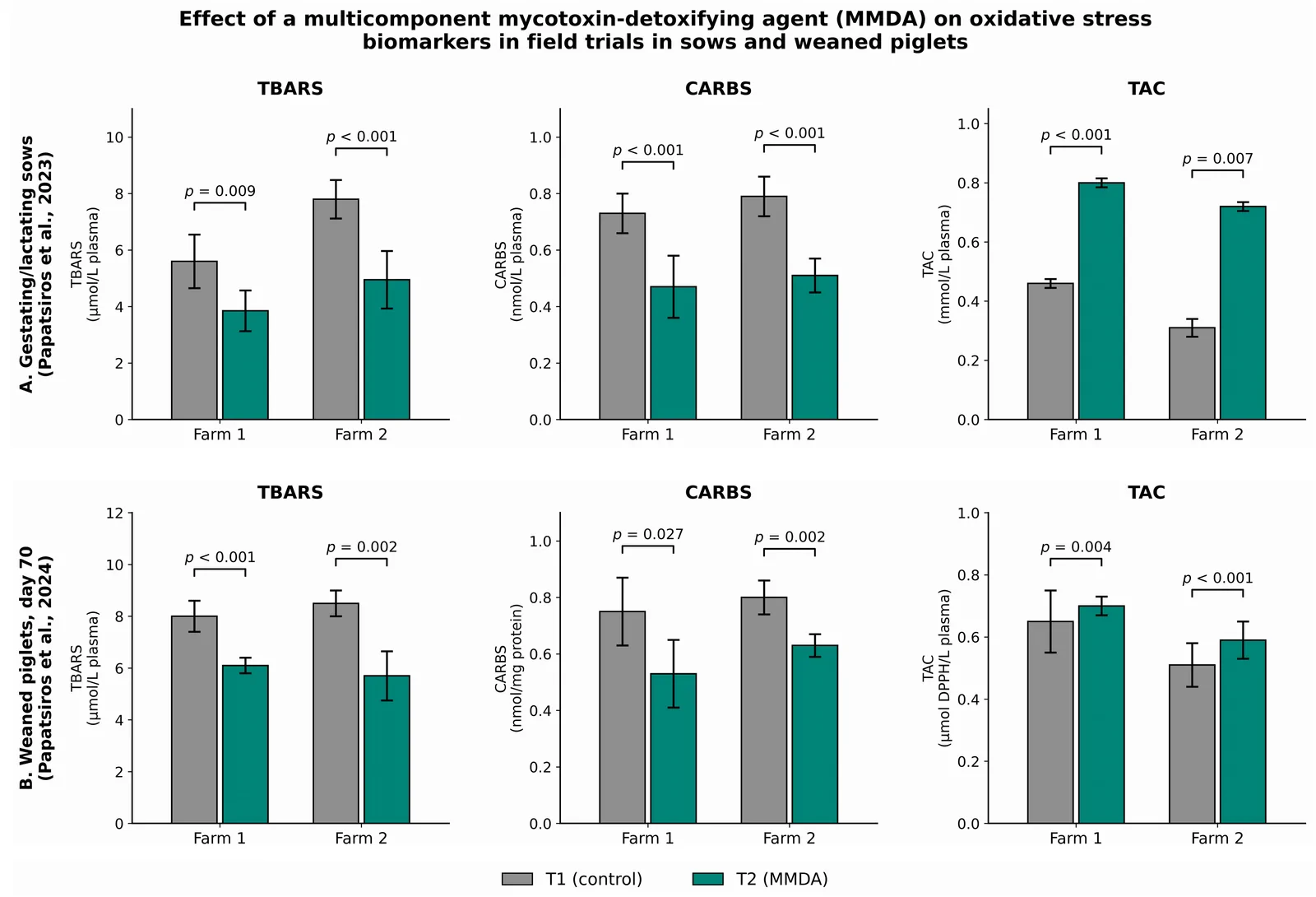
Effect of a multicomponent mycotoxin-detoxifying agent (MMDA) on plasma oxidative stress biomarkers (TBARS, CARBS, TAC) in field trials in (**A**) gestating/lactating sows and (**B**) weaned piglets (day 70), by farm. Bars represent mean ± SD for the contaminated-feed control group (T1, gray) and the MMDA-supplemented group (T2, teal). Data replotted from Papatsiros et al. [10,11].

Figure 3 summarizes the plasma oxidative stress biomarker findings reported in the authors’ own field trials in weaned piglets [11] and in gestating/lactating sows [10], illustrating the consistent reduction in TBARS and protein carbonyls (CARBS) and the concomitant increase in total antioxidant capacity (TAC) in the MMDA-supplemented (T2) groups relative to the contaminated-feed controls (T1) across both independent Greek commercial farm trials.

### 4.2. Evidence in Gestating and Lactating Sows

In a dual-farm field trial, Papatsiros et al. [10] enrolled 80 primiparous sows per farm (mean age 366 ± 3 days), randomized to contaminated feed alone (T1) or contaminated feed plus the MMDA (T2), administered from one month before farrowing through end of lactation. Statistically significant (*p* < 0.05) reductions in TBARS and CARBS alongside increases in TAC were observed in T2 sows at both farms. Clinical health parameters and reproductive performance were improved in T2 sows [10]. The reproductive performance improvements carry direct LCA relevance: sow reproductive efficiency determines FCR and thus the full LCA burden per kg pork. *Bacillus subtilis* ANSB01G culture in ZEN-challenged first-parity gilts partially normalized estrogen receptor concentrations and reproductive hormone profiles [42]. Zymdetox Z-2000 (ZEN lactonase) mitigated ZEN-induced reproductive toxicity in gilts through enzymatic lactone ring hydrolysis [32].

### 4.3. Evidence in Growing–Finishing Pigs and Food Safety

Papatsiros et al. [12] documented subclinical mycotoxicosis in commercial Greek finisher farms using slaughter-stage LC-MS/MS liver analysis, detecting FB1 at 2.91–8.30 µg/kg and OTA at 0.54 µg/kg without overt clinical signs, with associated histopathological hepatic lesions. In a controlled 42-day trial in 24 crossbred pigs challenged with three mycotoxins (AF ~0.5 mg/kg, FUM ~1.0 mg/kg, ZEN ~1.0 mg/kg), two commercial adsorbents at 1.5 and 3.0 kg/ton both ameliorated adverse growth and biochemical effects and reduced mycotoxin metabolite residues in liver and kidney, with effects more pronounced at 3.0 kg/ton [43]. Reducing tissue residues is directly aligned with CE food safety requirements: animal products from pigs fed circular by-product-inclusive diets must meet equivalent safety standards to conventionally fed animals.

**Figure 4:**
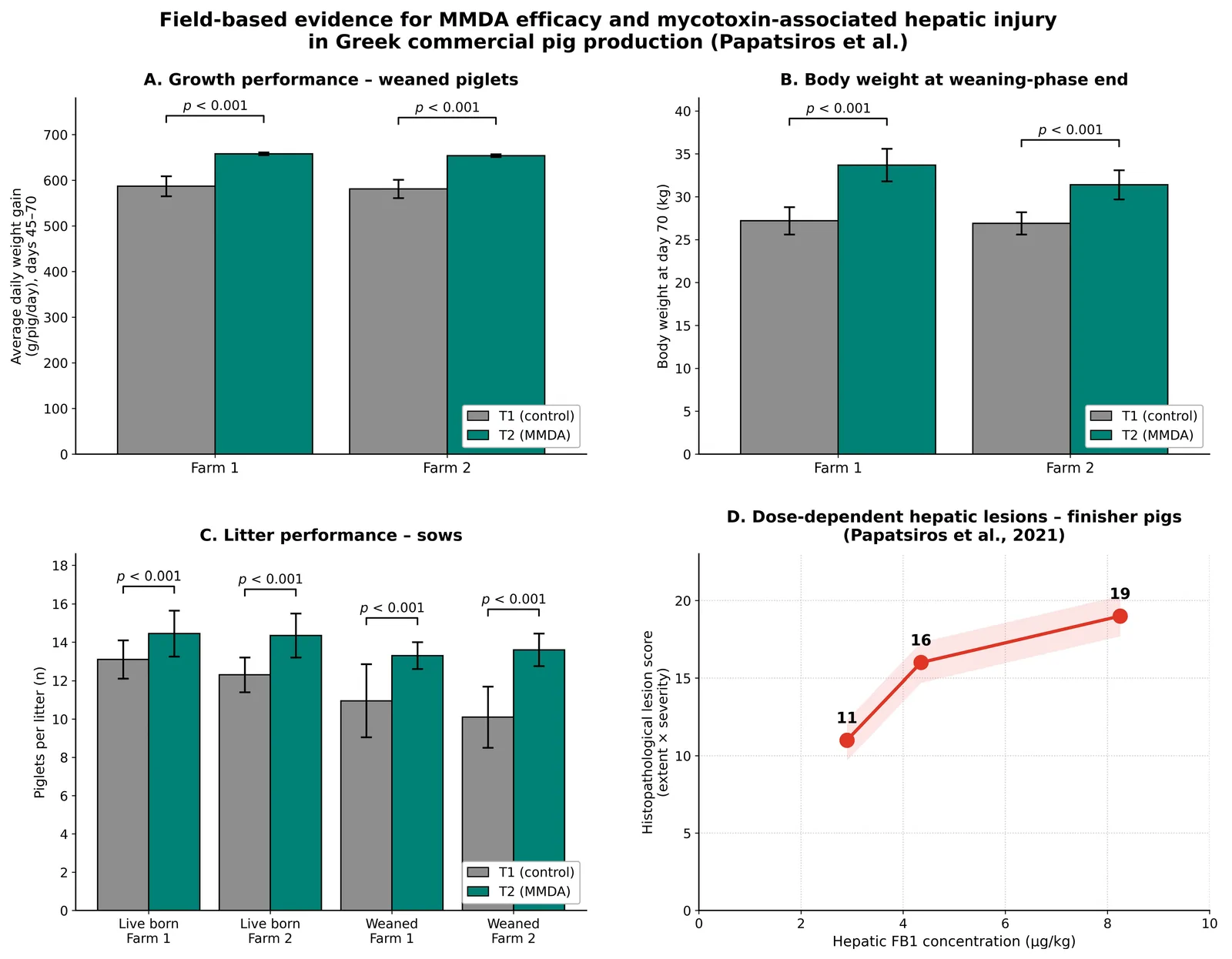
Field-based performance and histopathological evidence from Papatsiros et al. (**A**) Average daily weight gain (days 45–70) and (**B**) body weight at day 70 in weaned piglets, control (T1) vs. MMDA-supplemented (T2) groups, by farm [11]. (**C**) Litter performance (live-born and weaned piglets per litter) in sows, T1 vs. T2, by farm [10]. (**D**) Dose-dependent histopathological lesion score (extent × severity) in the liver of finisher pigs as a function of hepatic fumonisin B1 (FB1) concentration [12]. Bars/points represent mean ± SD; shaded band in (**D**) indicates ±1.5-point uncertainty envelope around the reported scores.

Figure 4 presents complementary own-data evidence on growth and reproductive performance from the same field trials, together with the dose-dependent hepatic lesion severity reported in naturally FB1-exposed finisher pigs, further supporting the biomarker framework summarized in Table 4.

### 4.4. Evidence in Boars

Boar data are relatively limited but highly relevant from a mechanistic standpoint, as the male reproductive axis itself is the target of both zearalenone and its metabolites. Because of their ability to mimic estrogens, ZEN, α-zearalenol and β-zearalenol disrupt spermatogenesis and sperm maturation; in vitro exposure of boar spermatozoa to ZEN and α-zearalenol leads to loss of motility, viability, membrane function and normal morphology, and combined exposure to DON and ZEN causes additive to synergistic adverse effects on boar semen characteristics [44]. In vivo, ZEN intake has been reported to reduce libido and serum testosterone levels, and to decrease testicular weight and spermatogenesis in young boars—parameters of clear economic importance in artificial insemination studs, where a single boar is responsible for the reproductive output of many sows [44,45]. Since boar spermatozoa have a high content of polyunsaturated fatty acids and low cytosolic antioxidant levels, they are particularly susceptible to the oxidative stress induced by mycotoxins, supporting the use of MMDA containing antioxidants.

## 5. Anti-Mycotoxin Agents in the Framework of Circular Economy

### 5.1. Circular Economy Principles in Livestock Feed

The circular economy (CE) replaces the linear take–make–dispose model with closed-loop systems that keep materials and resources in productive use at their highest value, guided by the principles of design for longevity, waste elimination, and regeneration of natural systems. In agrifood systems, CE translates into cascading biomass use: crops first for human food, then co-products and residues for animal feed, then organic matter for bioenergy and biofertilizers. This hierarchy of use, codified in the EU Waste Framework Directive and elaborated in the European Commission’s Circular Economy Action Plan (2020), explicitly identifies livestock production as a critical node in circular biomass value chains [39].

Pigs are natural CE converters, transforming food-chain co-products into high-quality animal protein with high biological efficiency. The Ellen MacArthur Foundation’s CE framework identifies monogastric livestock as uniquely suited to valorize low-grade biomass that is unsuitable for direct human consumption. From a quantitative perspective, the European agrifood industry generates an estimated 89 million tonnes of food waste annually, of which approximately 20–30% has potential value as feed ingredients following appropriate processing and safety verification [46]. The EU Farm to Fork Strategy, European Green Deal, and circular bioeconomy initiatives—projected to generate USD 7.7 trillion in economic value by 2030—all promote CE-consistent feed formulations as essential components of sustainable food system transformation [39]. The former foodstuffs (FF) regulatory framework (EU Regulation 576/2006) [25] and the by-products Regulation (EC) No 576/2006 [25] provide explicit legal pathways for incorporating food-industry co-products into pig feeds, yet the practical realization of these pathways is critically contingent on food safety assurance—of which mycotoxin management is a primary determinant [46].

The economic rationale for CE feed strategies in pig production is compelling. Cereal grains represent 60–75% of total feed cost in conventional pig diets, and their partial replacement with validated co-product streams can reduce feed cost by 10–25% depending on ingredient market volatility and formulation design [39]. Beyond direct cost reduction, CE feed strategies reduce competitive pressure on human-edible grain supply chains and decrease land-use intensity per unit of pork produced—both critical metrics in LCA-based sustainability assessments. The circular bioeconomy policy framework thus creates a convergent incentive structure in which mycotoxin management becomes simultaneously a veterinary necessity, an economic optimization lever, and an environmental sustainability requirement.

### 5.2. Mycotoxin Contamination as a Barrier to CE Feed Systems

The by-products most attractive for CE feed inclusion—distillers’ dried grains with solubles (DDGS), brewers’ grains, maize gluten feed, cereal screenings, wheat bran, rice bran, and former foodstuffs—disproportionately concentrate mycotoxins during processing, creating a fundamental paradox at the intersection of resource efficiency and food safety. Ethanol fermentation concentrates Fusarium toxins approximately threefold in DDGS, since mycotoxins are not degraded during fermentation and partition into the solid fraction [8]. Similarly, wet milling of maize concentrates DON and ZEN in gluten fractions, and cold-pressing of oil seeds concentrates OTA in press cakes. Systematic reviews confirm that mycotoxin contamination occurs even in dried matrices and constitutes a major constraint on reliable by-product valorization [7]. A comprehensive global survey of CE-relevant feed ingredients documented DON in 72% of DDGS samples at mean concentrations of 1.84 mg/kg—above the EU guidance value for complete pig feeds—and ZEN in 61% of samples [8]. Unintended food safety impacts of circular agricultural practices explicitly identify mycotoxins as primary risks from reintroduction of agricultural residues into feed chains, particularly under the co-contamination conditions prevalent in commercial settings [9]. Without effective anti-mycotoxin agents, expanding CE-consistent feed formulations would carry unacceptable animal health and food safety risks that would undermine the regulatory and market viability of circular feed strategies.

The mycotoxin concentration effect of CE processing is ingredient- and toxin-specific and must be incorporated into feed safety risk models. DDGS from maize bioethanol production shows the most extreme concentration effect for DON (2.5–3.5×), ZEN (2.0–3.0×), and fumonisins (2.0–3.5×), reflecting their water-insolubility and resistance to fermentation [8,9]. Brewers’ spent grain, while primarily a barley matrix with lower intrinsic Fusarium contamination, can accumulate OTA from Penicillium contamination during moist storage. Maize gluten feed concentrates fumonisins and aflatoxins in the protein-rich fraction. Former foodstuffs present the most heterogeneous contamination profile due to their diverse origin: baked goods, pasta, and confectionery residues may carry AFB1, OTA, or Fusarium toxins depending on their cereal ingredient origin and storage history [8]. The regulatory classification of these ingredients as animal feed under EU Regulation 767/2006 [25] does not automatically guarantee conformity with mycotoxin guidance values when sourced from CE streams, necessitating systematic lot-by-lot mycotoxin testing as a prerequisite for responsible CE feed formulation.

The co-contamination problem is particularly acute in CE feed systems. Multi-mycotoxin surveys of CE-relevant ingredients routinely detect two to five mycotoxins in the same sample [8]. This co-contamination pattern is toxicologically significant: DON and ZEN co-exposure produces additive or synergistic intestinal barrier disruption; AFB1 and FB1 co-exposure synergistically impairs hepatic function; and OTA combined with AFB1 amplifies nephrotoxic injury in pig kidneys [24,25]. The practical implication is that individual mycotoxin concentration factors applied in isolation underestimate the actual risk to pigs consuming CE diets, because the relevant safety threshold for any single toxin is effectively reduced in the presence of co-contaminants. This biological reality reinforces the requirement for multi-target anti-mycotoxin strategies rather than single-agent approaches in CE feeding systems. Climate change is projected to expand *Aspergillus* ecological range into temperate European regions, amplifying the mycotoxin management burden in CE-consistent feed systems that incorporate co-products, with modeling projections indicating a 20–30% increase in aflatoxin risk in Southern European maize by 2050 under RCP8.5 climate scenarios [13].

### 5.3. Anti-Mycotoxin Agents as CE Enablers

Anti-mycotoxin agents contribute to CE objectives through multiple distinct and quantifiable mechanisms. First, by enabling the safe circular use of borderline-contaminated cereal fractions and by-product streams that would otherwise be diverted to non-feed uses or incineration, they directly expand the feedstock base available to pig producers operating CE formulation strategies. This valorization function is economically significant: at a conservative estimate of 150 EUR/tonne for displaced DDGS relative to primary maize, enabling a 10% substitution in a 3-tonne/sow/year feed program represents approximately 45 EUR/sow/year in direct feed cost reduction, not accounting for any performance co-benefits. Second, anti-mycotoxin agents reduce feed waste from DON-induced feed refusal and emesis, which represent total metabolic dead-weight losses in CE terms: feed that is refused or vomited has consumed all upstream agricultural and processing resources with zero conversion to animal products. DON-induced feed intake reductions of 10–30% documented in challenge studies represent proportional upstream resource waste across land, water, fertilizer, and energy inputs [6,7]. Third, they reduce slaughter condemnations from organ pathology—representing total loss of all resources invested in the condemned animal across its entire production cycle. Fourth, by reducing mycotoxin tissue carry-over (AFB1, OTA, FB1), they protect the human food chain and enable CE pork products to meet equivalent food safety standards to conventionally produced pork [8,46].

The CE alignment of MMDA ingredient sourcing itself deserves systematic attention. Many key MMDA components are themselves products of circular or resource-efficient supply chains. Bentonite and sepiolite are geological resources extracted with relatively low energy intensity and available in substantial quantities in Southern Europe (Spain, Greece, Turkey), enabling short-supply-chain sourcing for European pig producers. Curcumin derives from *Curcuma longa* agricultural processing, with the rhizome residue after curcuminoid extraction reusable as feed material; silymarin is extracted from *Silybum marianum* seeds cultivable on marginal land unsuitable for food crops, adding no additional pressure on arable land. Yeast cell wall fractions—providing β-1,3/1,6-glucans, mannoproteins, and mannan-oligosaccharides—are co-products of *Saccharomyces cerevisiae* fermentation in the brewing and bioethanol industries; hydrolyzed yeast is produced from the same spent yeast biomass through enzymatic autolysis, converting what was previously a fermentation waste stream into a high-value feed bioactive [25]. This convergence of CE-aligned ingredient sourcing with CE-enabling functional activity positions MMDAs as exemplary circular economy technologies at two levels: their material inputs embody circular principles, and their functional outputs enable circular feed systems.

A critical but underappreciated CE-enabling function of anti-mycotoxin agents is the reduction in whole-enterprise resource losses attributable to sow reproductive failure. In CE accounting, a sow that fails to farrow or produces a reduced litter due to ZEN or DON exposure represents a complete waste of all feed, housing, veterinary, and energy resources invested in that animal from weaning through the failed reproductive event. Reproductive failure rates of 5–15% attributable to subclinical mycotoxicosis—documented under field conditions in commercial European herds [12]—translate to proportional increases in the whole-enterprise resource intensity per unit of pork produced. MMDA supplementation of sows from pre-farrowing through end of lactation, as evaluated by Papatsiros et al. [10], directly addresses this CE resource efficiency gap by protecting the productive integrity of the reproductive unit and thereby reducing the upstream resource cost per kilogram of pork produced at the enterprise level.

### 5.4. Integrating CE Feed Safety into Regulatory and Certification Frameworks

The regulatory framework governing CE feed safety in the EU is multi-layered and evolving. EU Regulation 767/2009 on the placing on the market of feed specifies labeling and composition requirements for feed materials and compound feeds, including by-products and former foodstuffs. Commission Recommendation 2006/576/EC [25] sets indicative maximum mycotoxin levels for complete pig feeds, but these guidance values apply to finished feeds and do not prescribe how producers should manage the elevated contamination risk inherent in CE feed ingredients before inclusion. The gap between ingredient-level mycotoxin concentrations in CE streams and finished feed guidance values must be managed through a combination of lot-testing, blending strategies, and anti-mycotoxin agent supplementation [7,36]. The EU Farm to Fork Strategy’s 2030 ambition to reduce food loss and waste by 50% and to increase circularity in food systems will inevitably intensify the use of CE feed ingredients, placing greater regulatory pressure on the mycotoxin management infrastructure of the swine sector. The authorization of anti-mycotoxin feed additives under Regulation (EC) No 576/2006 [25] thus acquires a dual significance: beyond its traditional animal health protection rationale, it increasingly functions as an enabler of regulatory compliance for CE-consistent feed formulations.

Private certification and quality assurance schemes increasingly address CE feed safety at the supply chain level. The GMP+ Feed Safety Assurance scheme, the QS Quality and Safety system all include mycotoxin monitoring requirements for feed ingredients. However, none of these schemes currently specifies requirements for anti-mycotoxin agent supplementation as a mandatory safeguard when CE feed ingredients exceeding defined threshold concentrations are used—a gap that industry and regulatory stakeholders are beginning to address. From a One Health perspective, the food safety implications of CE feeding extend beyond the farm gate: EFSA’s assessments of OTA and fumonisins specifically identify pork and pork products as significant contributors to human mycotoxin dietary exposure, and flag CE feeding practices involving concentrated by-products as an emerging risk factor requiring ongoing monitoring [8]. The quantification of the CE safety enabling value of anti-mycotoxin agents remains an unmet methodological need. An integrated CE-LCA modeling framework incorporating mycotoxin concentration factors for CE feed ingredients, dose–response relationships between mycotoxin exposure and performance endpoints, anti-mycotoxin agent efficacy parameters from field-validated studies such as Papatsiros et al. [10,11], and upstream resource costs per unit of feed and pork produced would provide the quantitative basis for CE sustainability claims in swine production—a priority research direction identified in Section 7 of this review [43].

## 6. Life Cycle Assessment of Pig Production and the Role of Feed Additives

### 6.1. LCA Methodology and Environmental Hotspots

LCA quantifies environmental impacts across the full lifecycle from raw material extraction through end-of-life. In pig production, the cradle-to-gate system boundary encompasses upstream feed production (crop cultivation, fertilizer manufacture, processing, transport), on-farm processes (rearing, manure management, energy), and downstream (slaughter, processing). A systematic review of 74 LCA studies from 27 countries (2005–2023) reported pork GWP of 3.9–10 kg CO_2_-eq/kg live weight, with feed production contributing 22–95% (mean 59 ± 18%) and FCR as the primary driver across GWP, AP, EP, and LO (correlation > 0.95) [6]. German cradle-to-farm-gate LCA studies confirmed feed dominance across all impact categories [7]. Table 5 presents the integration of LCA impact categories with mycotoxin effects and expected MMDA benefits.

### 6.2. Quantifiable LCA Benefit of FCR Improvement

The strong FCR–LCA correlation means any feed additive improving FCR produces simultaneous proportional reductions across all environmental impact categories. An LCA of *Saccharomyces cerevisiae* probiotic supplementation in dairy cows (Actisaf Sc 47) demonstrated a 2.9% annual carbon footprint reduction alongside proportional reductions in land use (2.05%), water use (2.47%), acidification (2.28%), freshwater eutrophication (2.18%), and marine eutrophication (2.14%), with the additive’s own production burden representing only 0.005–0.016% of total carbon footprint [47,48,49]. This provides a methodological template and order-of-magnitude benchmark for MMDA LCA benefit calculations in pigs. LCA of specialty feed ingredients in pig systems across Europe, North America, and South America confirmed that FCR improvements from feed additives produce proportional environmental co-benefits across all impact categories [48].

## 7. Anti-Mycotoxin Agents, Circular Economy, and LCA in Pig Production: Alignment with the UN Sustainable Development Goals

The United Nations 2030 Agenda for Sustainable Development established 17 Sustainable Development Goals (SDGs) as a universal framework for addressing the interconnected challenges of poverty, health, environmental sustainability, and economic equity. Livestock production systems, including intensive pig production, intersect with multiple SDGs simultaneously—as sources of both risk and opportunity. The integrated application of anti-mycotoxin agents within circular economy (CE) feed strategies and life cycle assessment (LCA)-guided production management creates a coherent cluster of SDG co-benefits that positions effective mycotoxin management as a concrete, farm-level contribution to the global sustainability agenda. Table 6 summarizes the principal SDG linkages of this integrated framework in the pig sector.

### 7.1. SDG 2 (Zero Hunger) and SDG 3 (Good Health and Well-Being)

SDG 2 calls for ending hunger, achieving food security, improving nutrition, and promoting sustainable agriculture. Mycotoxin contamination of pig feed directly undermines food security at two levels: it reduces the efficiency of conversion of plant-based calories into animal protein (through FCR deterioration), and it introduces hazardous residues—aflatoxin M1 analogs, OTA, and fumonisin metabolites—into pork products consumed by human populations. Anti-mycotoxin agents address both failure modes simultaneously, protecting feed-to-food conversion efficiency and reducing mycotoxin carry-over into the human food chain [2,12]. Within CE feeding systems, their enabling role expands access to affordable protein by reducing feed costs through safe by-product valorization—a contribution directly aligned with SDG 2’s target of sustainable food system transformation. SDG 3 (Good Health and Well-Being) is addressed through the One Health dimension of mycotoxin management: OTA is classified as an IARC Group 2B carcinogen and accumulates in pork products; AFB1 is IARC Group 1 and carries over as AFM1 in edible tissues; fumonisin B1 is associated with esophageal cancer risk in human populations [25]. By reducing tissue residue burdens in pigs fed CE-inclusive diets—as documented by Papatsiros et al. [12] using LC-MS/MS slaughter-stage analysis—anti-mycotoxin agents make a measurable contribution to consumer health protection, directly relevant to SDG 3 Target 3.d on strengthening food safety capacity.

### 7.2. SDG 12 (Responsible Consumption and Production) and SDG 13 (Climate Action)

SDG 12 (Responsible Consumption and Production) encompasses targets on sustainable management of natural resources, halving per capita global food waste (Target 12.3), and achieving environmentally sound management of chemicals and waste (Target 12.4). The CE framework operationalized in pig production through MMDA-enabled by-product valorization directly advances SDG 12.3: DDGS, brewers’ grains, cereal screenings, and former foodstuffs that would otherwise be downgraded or landfilled are safely incorporated into pig diets, reducing agrifood system waste and closing biomass loops. By preventing DON-induced feed refusal and emesis, anti-mycotoxin agents additionally reduce on-farm feed waste—a direct SDG 12.3 contribution quantifiable in tonnes of feed saved per sow per year. SDG 12.4 (sound chemical management) is addressed through the reduction in mycotoxin residue burdens in pork products and the responsible use of scientifically validated, EFSA-authorized feed additives rather than unregulated mycotoxin management approaches.

SDG 13 (Climate Action) is addressed through the LCA pathway described in Section 5. Feed production contributes 22–95% (mean 59 ± 18%) of pork’s greenhouse gas (GHG) footprint, with FCR as the primary driver [6]. Mycotoxin-induced FCR deterioration of 5–15% from DON alone translates directly into proportional increases in global warming potential (GWP), eutrophication potential (EP), and acidification potential (AP) per kg of pork produced. MMDA supplementation that restores FCR to baseline generates quantifiable GHG co-reductions aligned with SDG 13 Target 13.2 (integrating climate measures into national policies, strategies, and planning). At the system level, CE-enabled feed strategies that replace virgin cereal inputs with co-product streams further reduce the land-use and fertilizer-derived N_2_O emissions embedded in pig feed, amplifying the SDG 13 contribution beyond what FCR improvement alone achieves. The LCA precedent from dairy cow probiotic supplementation—2.9% annual carbon footprint reduction from a single feed additive [49]—provides an order-of-magnitude benchmark for the SDG 13 relevance of MMDA use at scale across the European pig sector.

### 7.3. SDG 15 (Life on Land), SDG 6 (Clean Water), and SDG 8 (Decent Work and Economic Growth)

SDG 15 (Life on Land) encompasses targets on sustainable land use, halting biodiversity loss, and combating land degradation. The land occupation (LO) impact category in pig LCA—driven primarily by feed crop area requirements—is directly reduced when FCR improves and when CE feed strategies substitute crop-based ingredients with co-products. LO reductions of 5–10% achievable through combined FCR improvement and CE ingredient substitution translate into proportional reductions in the pressure on natural habitats, deforestation drivers (particularly soy-associated), and biodiversity loss attributed to pig feed production [6,48]. SDG 6 (Clean Water) is addressed through the eutrophication potential (EP) pathway: mycotoxin-induced FCR deterioration increases nitrogen and phosphorus excretion per kg of pig produced, intensifying the nutrient loading of water bodies surrounding pig farms. MMDA-mediated FCR restoration reduces N and P excretion proportionally, contributing to SDG 6 Target 6.3 (improving water quality by reducing pollution) and Target 6.6 (protecting water-related ecosystems).

SDG 8 (Decent Work and Economic Growth) is addressed through the economic dimension of mycotoxin management in the pig sector. Mycotoxin-induced losses—estimated at USD 1.4 billion annually in the United States alone from the three principal mycotoxins [2]—represent direct economic destruction within agricultural value chains that disproportionately affects smallholder and medium-scale pig producers with limited capacity for feed quality management. Effective MMDA-based mycotoxin management protects farm income, reduces veterinary expenditure, prevents slaughter condemnations, and enables safe participation in CE feed markets—all contributing to SDG 8 Target 8.2 (higher economic productivity through technological upgrading) and Target 8.3 (promoting decent work and economic growth in agricultural value chains). The circular bioeconomy framework projected to generate USD 7.7 trillion by 2030 [39] additionally represents a job-creation opportunity in bio-based ingredient processing, CE logistics, and feed safety testing—economic activities that expand when mycotoxin management enables reliable CE feed valorization.

### 7.4. The One Health Framework as an SDG Integration Mechanism

**Figure 5:**
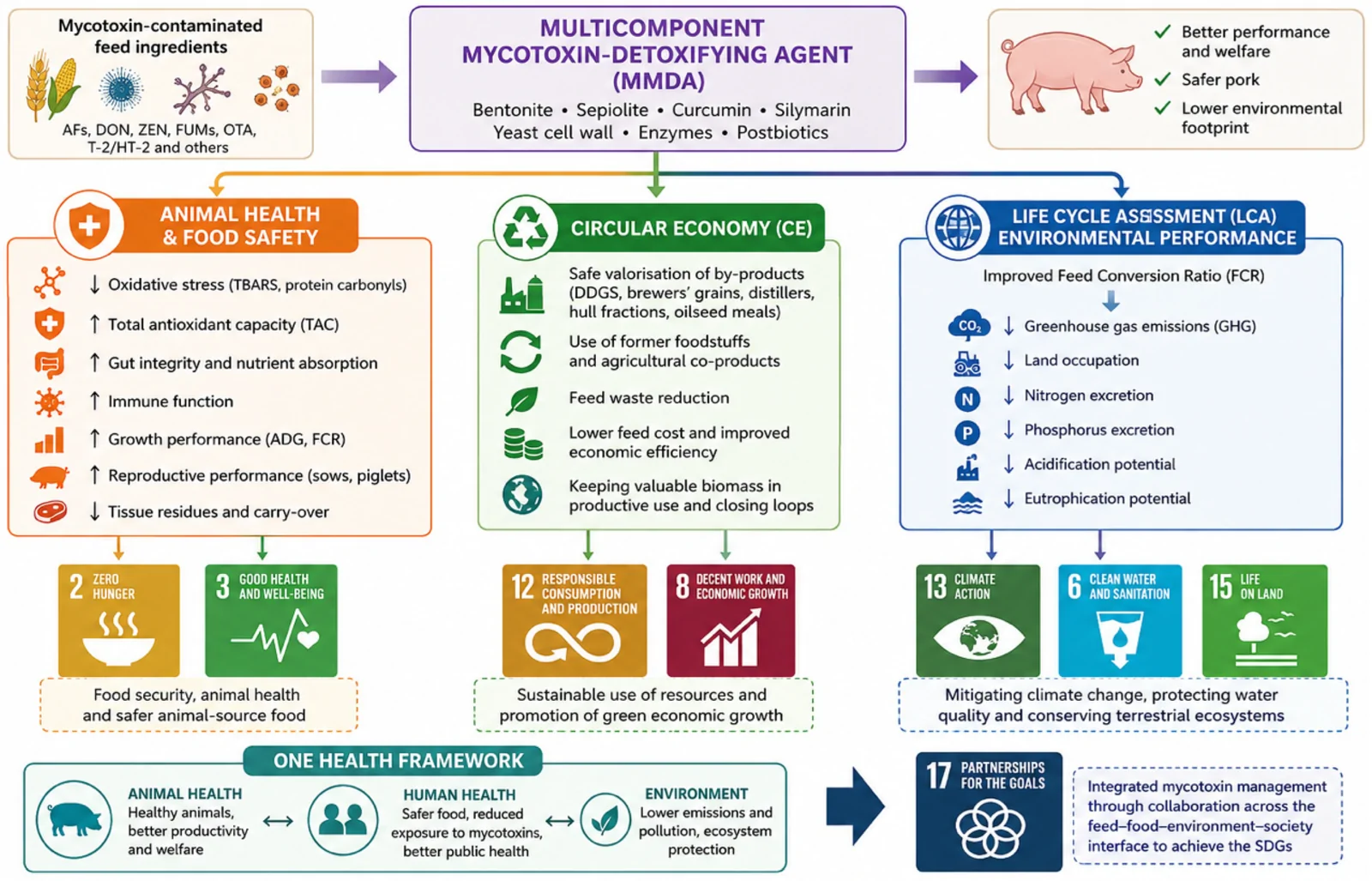
Integrated contribution of anti-mycotoxin agents to the United Nations Sustainable Development Goals (SDGs) through circular economy (CE), life cycle assessment (LCA), and One Health in pig production. MMDA supplementation improves animal health and food safety, enables circular feed strategies, and reduces environmental impacts. These combined benefits support SDGs 2, 3, 6, 8, 12, 13, 15, and 17.

The One Health framework—recognizing the inextricable interconnection between animal health, human health, and ecosystem health—provides the conceptual architecture that integrates the individual SDG contributions described above into a coherent system-level perspective. Mycotoxin management in pig production is inherently a One Health intervention: it simultaneously protects swine welfare and productivity (animal health), reduces human dietary exposure to IARC-classified carcinogens via pork products (human health), and reduces the environmental footprint of feed production and nutrient excretion (ecosystem health). This tripartite impact structure maps precisely onto the SDG architecture, which similarly recognizes that health, economic, and environmental objectives are inseparable. The Quadripartite One Health Joint Plan of Action (2022–2026), co-developed by FAO, UNEP, WHO, and WOAH, explicitly identifies food safety—including mycotoxin control in livestock feed chains—as a priority One Health action area [5]. Positioning MMDA-based mycotoxin management as a One Health SDG contribution, backed by field-validated biomarker evidence from commercial pig production [10,11,12], provides pig producers, veterinarians, feed industry stakeholders, and policymakers with a shared conceptual and policy language for integrating farm-level mycotoxin management decisions into national and international sustainability commitments [Figure 5].

## 8. Regulatory Framework

The EU framework for anti-mycotoxin feed additives rests on Regulation (EC) No 1831/2003, defining the functional group of ‘substances for reduction in contamination of feed by mycotoxins.’ Authorization requires an EFSA scientific opinion confirming efficacy, target animal safety, user safety, consumer safety, and environmental safety. Maximum recommended mycotoxin levels in complete pig feeds are set under Commission Recommendation 2006/576/EC: DON 0.9 mg/kg, ZEN 0.1 mg/kg (sows)/0.25 mg/kg (others), OTA 0.05 mg/kg, AFB1 0.01 mg/kg, FUM (FB1 + FB2) 5 mg/kg (sows)/1 mg/kg (piglets) [26]. EFSA risk assessments for OTA, FUM, DON, and ZEN increasingly incorporate CE feed scenarios and cumulative exposure considerations. The EU Farm to Fork Strategy reinforces the necessity for validated anti-mycotoxin strategies as prerequisite conditions for CE-consistent feed formulations incorporating co-products.

## 9. Critical Appraisal, Limitations, and Future Research Directions

- LCA integration gap: No published pig LCA has formally modeled anti-mycotoxin agent use as a functional input. Developing dedicated models incorporating FCR and reproductive improvements from field-validated MMDAs—including data from Papatsiros et al. [10,11]—is the most immediate research priority.
- Outcome standardization: Heterogeneity in biomarker panels, challenge doses, and production stages limits cross-study comparability. The consistent TBARS–CARBS–TAC panel deployed by Papatsiros et al. [10,11] is a commendable standardization effort enabling future meta-analytic integration.
- Dose–response non-linearity: As demonstrated by Raj et al. [47] and corroborated by Papatsiros et al. [10], dose–response relationships of MMDAs are not consistently linear across all parameters. Future studies should employ formal dose–response modeling using mixed-effects regression frameworks.
- Nutritional antagonism: Mineral adsorbents may co-adsorb fat-soluble vitamins (A, D, E, K) and trace minerals (Se, Zn) at higher inclusion rates. This opportunity cost should be incorporated into net LCA benefit calculations.
- Long-term and lifecycle data: Most intervention trials span 28–70 days. Evidence for sustained MMDA efficacy over full production cycles or across multiple consecutive parities is absent and required for whole-lifecycle LCA modeling.
- MMDA production LCA: The environmental footprint of MMDA production itself (mining, phytogenic extraction, yeast fermentation and drying) should be formally quantified for net LCA benefit calculation, following the dairy probiotic LCA precedent [49].
- CE feed safety quantification: Comparative studies assessing tissue residue accumulation in pigs fed CE-formulated diets with and without anti-mycotoxin agents are needed to quantify the food safety enabling role of MMDAs in circular systems.
- Climate change scenarios: As mycotoxin contamination profiles shift with climate change, MMDA efficacy profiles and LCA benefit calculations should incorporate dynamic contamination scenarios aligned with IPCC projections.

## 10. Conclusions

Anti-mycotoxin agents in pig production occupy a uniquely multidimensional role at the intersection of animal health, food safety, and environmental sustainability. The clinical evidence base—spanning adsorbents, biotransforming preparations, phytogenic antioxidants, and advanced MMDAs—has grown substantially across all major mycotoxin categories and production stages. Studies conducted in commercial grower-finisher pigs, sows, and post-weaning pigs have provided a coherent in vivo approach to the subclinical deposition of toxicant residues in tissues, the assessment of the efficacy of anti-mycotoxic agents under multi-mycotoxic natural exposure, and the positive impact on antioxidants and growth performance, via a reliable combination of TBARS, CARBS, and TAC biomarkers, as a methodological framework for all production phases. In our view, however, the field is currently hampered by short trial periods, heterogeneous exposure conditions, and limited information on breeding boars and complete production cycles.

Within the CE framework, anti-mycotoxin agents function as enabling technologies that allow borderline-contaminated cereal fractions, food-industry by-products, and former foodstuffs to be safely incorporated into pig diets, reducing dependence on virgin cereal inputs, preventing biomass loss to waste, and protecting the human food chain from mycotoxin residue carry-over. The natural or co-product origin of key MMDA ingredients aligns with CE material sourcing principles.

Within the LCA framework, FCR improvements documented across MMDA field and controlled trials generate quantifiable co-reductions across GWP, AP, EP, land use, and water use through well-established FCR–LCA sensitivity relationships. Developing dedicated pig anti-mycotoxin LCA models integrating field-validated performance data from natural contamination scenarios represents the most impactful research frontier at the intersection of veterinary medicine and environmental sustainability in swine production.

## 11. Materials and Methods

This study is an integrative (narrative) review. To ensure the reproducibility and transparency of the evidence base, the literature search was conducted in accordance with the reporting criteria outlined in the PRISMA 2020 guidelines [49] for narrative synthesis. The databases PubMed, Scopus, Web of Science and Google Scholar were searched for records from January 2000 to June 2026 using combinations of the following keywords: “mycotoxin”, “pig” OR “swine”, “anti-mycotoxin agent”, “adsorbent”, “biotransformation”, “phytogenic”, “multicomponent mycotoxin-detoxifying agent”, “oxidative stress”, “circular economy” and “life cycle assessment”. Only peer-reviewed primary studies and reviews in English relevant to mycotoxin toxicology, anti-mycotoxin agents and the circular economy/life cycle assessment of pig production were included. Conference abstracts and non-peer-reviewed sources were excluded. Priority was given to in vivo and field studies in pigs and the most recent surveillance and regulatory data; the reference lists of the selected articles were searched for additional information.

## Figures and Tables

**Table 1:** Reported occurrence of the principal mycotoxins in swine-relevant cereals and complete feeds across recent European and global surveys. AFB1, aflatoxin B1; DON, deoxynivalenol; ZEN, zearalenone; FB1, fumonisin B1; OTA, ochratoxin A; DDGS, distillers’ dried grains with solubles; CE, circular economy.

Region/Matrix	Survey Period	Predominant Mycotoxins	Reported Occurrence and Co-Contamination	Ref.
Maize, Serbia	2021–2023	AFB1, fumonisins, DON, ZEN	AFB1 above the 5 µg/kg limit in 73.2% of samples in the 2022 drought year; fumonisins the most prevalent contaminant; frequent co-contamination	[13]
Complete pig feeds, Southern Romania	2021–2024	DON, ZEN, fumonisins, OTA	Sub-chronic multi-mycotoxin co-exposure the predominant commercial exposure pattern	[14]
Global feed survey (>100 countries)	10-year	DON, fumonisins, ZEN, aflatoxins	60–80% of samples contained at least one mycotoxin; DON and ZEN among the most frequent; co-contamination common	[5,6]
CE by-products (DDGS), global	systematic review	DON, ZEN, fumonisins	DON in 72% of DDGS samples (mean 1.84 mg/kg) and ZEN in 61%; ~3-fold concentration during processing	[10,11]
Pig liver at slaughter, Greece	field study	FB1, OTA	FB1 2.91–8.30 µg/kg and OTA 0.54 µg/kg detected with hepatocellular lesions (subclinical exposure)	[12]

**Table 2:** Principal mycotoxins of concern in pig production: EU regulatory thresholds, primary toxic targets, and key sustainability implications.

Mycotoxin	Producer Fungus	EU Max (Complete Pig Feed)	Primary Porcine Target	Key Toxic Mechanism	Sustainability/CE Implication
AFB1	*Aspergillus* spp.	0.01 mg/kg	Liver, immune system	CYP450 epoxide → DNA adducts	Tissue residues (AFM1) risk pork safety in CE systems
DON	*Fusarium graminearum*	0.9 mg/kg	Intestine, immune system	Ribotoxic stress → barrier disruption	FCR ↑ 5–15% → GWP, EP, AP ↑ proportionally [6,7]
ZEN	*Fusarium graminearum*	0.1 mg/kg (sows)	Reproductive system	ER-α binding → estrogenism, reproductive failure	Reproductive failure inflates FCRwe → full LCA burden ↑
FB1 + FB2	*Fusarium verticillioides*	5 mg/kg (sows)	Lungs, liver, gut	Ceramide synthase inhibition	FB1 liver residues confirmed in field pigs [13]; CE by-products concentrate FUMs
OTA	*Aspergillus/Penicillium*	0.05 mg/kg	Kidney (proximal tubule)	Nephrotoxicity; t½ ~88.8 h in pigs	Pork OTA carry-over; IARC Group 2B carcinogen [26]
T-2/HT-2	*Fusarium sporotrichioides*	No EU limit set	Skin, hematopoietic	Ribosomal binding; epoxide toxicity	Synergistic tissue residue deposition with ZEN [9]

AFB1, aflatoxin B1; DON, deoxynivalenol; ZEN, zearalenone; FB1/FB2, fumonisin B1/B2; OTA, ochratoxin A; ER-α, estrogen receptor-alpha; FCR, feed conversion ratio; FCRwe, whole-enterprise FCR; GWP, global warming potential; EP, eutrophication potential; AP, acidification potential; CE, circular economy; LCA, life cycle assessment. EU thresholds from Commission Recommendation 2006/576/EC [26].

**Table 3:** Principal phytogenic anti-mycotoxin agents evaluated in monogastric species: botanical source, main active principles, and predominant protective mechanisms. SOD, superoxide dismutase; CAT, catalase; GSH-Px, glutathione peroxidase; CYP450, cytochrome P450; AFB1, aflatoxin B1; OTA, ochratoxin A; ZEN, zearalenone; DON, deoxynivalenol; FB1, fumonisin B1; AF, aflatoxins; FUM, fumonisins.

Phytogenic Agent	Botanical Source	Main Active Principle(s)	Principal Protective Mechanism	Target Mycotoxins/Ref.
Curcumin	*Curcuma longa*	Curcuminoids (curcumin, demethoxy- and bisdemethoxycurcumin)	Radical scavenging; Nrf2/ARE induction of SOD, CAT and GSH-Px; CYP450 inhibition; NF-κB downregulation	AFB1, OTA, ZEN, DON, FB1 [30,35,38]
Silymarin	*Silybum marianum*	Silybin/silibinin, silychristin, silydianin	Hepatocyte membrane stabilization; glutathione preservation; ribosomal RNA synthesis and hepatocyte regeneration	OTA, AFB1, FB1 [35]
Grape-seed/pomace extract	*Vitis vinifera*	Proanthocyanidins, resveratrol	Radical scavenging; metal-ion chelation; reduced toxin bioactivation	AFB1, FB1 [35]
Green-tea extract	*Camellia sinensis*	Catechins (epigallocatechin-3-gallate)	Radical scavenging; Nrf2 activation	AFB1, OTA [35]
Olive-pomace extract	*Olea europaea*	Hydroxytyrosol, oleuropein	Radical scavenging; anti-inflammatory	AF, FUM [35]
Liquorice extract	*Glycyrrhiza glabra*	Glycyrrhizic acid (triterpenoid saponin)	Anti-inflammatory; gut-microbiota modulation; intestinal recovery	DON [40]
*Spirulina*	*Spirulina platensis*	Phycocyanin, β-carotene, tocopherols	Radical scavenging; SOD/CAT induction; anti-inflammatory	AFB1 [30]

**Table 4:** Biomarker framework for evaluating antimycotoxin agent efficacy in pigs, with relevance to CE and LCA sustainability objectives.

Biomarker Domain	Specific Parameters	Mycotoxin Target	CE/LCA Relevance	Key Porcine Studies
Oxidative stress	TBARS (µmol/L), CARBS (nmol/L), TAC (mmol/L), MDA, GSH, SOD, GSH-Px	FB1, AFB1, DON, ZEN	Health proxy; correlates with FCR impairment	Papatsiros et al. [11,12]; Deng et al. [31]
Growth performance	ADG, FCR, ADFI, BW, mortality	DON, ZEN, FUM, AF	FCR directly drives LCA GWP, EP, AP, LO [5,6]	Papatsiros et al. [11]; Raj et al. [29]
Tissue residues	Mycotoxin concentrations in liver, kidney, muscle (LC-MS/MS)	DON, ZEN, FB1, OTA, AFB1	Food safety in CE pork products; One Health	Papatsiros et al. [12]; Raj et al. [29]
Reproductive performance	Piglets born alive, litter weight, farrowing rate, vulva score, hormones	ZEN, DON (periparturient)	Determines FCRwe → full pork LCA burden	Papatsiros et al. [11]; Zhao et al. [34]
Intestinal integrity	Villus height:crypt depth, ZO-1, occludin, claudin-1, TEER, sIgA	DON, FB1	Nutrient absorption efficiency → FCR	Deng et al. [37]; Xu et al. [40]
Immune response	TNF-α, IL-1β, IL-6, T-cell subsets, IgA, IgG	AF, DON, ZEN	Disease burden → veterinary costs, mortality → LCA	Kim et al. [36]; Xu et al. [40]
Histopathology	Hepatocyte vacuolation, tubular degeneration, villi morphology (H&E)	FB1, OTA, AFB1	Condemnation risk at slaughter → resource loss	Papatsiros et al. [11,12]

TBARS, thiobarbituric acid reactive substances; CARBS, protein carbonyls; TAC, total antioxidant capacity; MDA, malondialdehyde; GSH, glutathione; SOD, superoxide dismutase; GSH-Px, glutathione peroxidase; ADG, average daily gain; FCR, feed conversion ratio; ADFI, average daily feed intake; BW, body weight; TEER, transepithelial electrical resistance; sIgA, secretory immunoglobulin A; TNF-α, tumor necrosis factor-alpha; IL, interleukin; H&E, hematoxylin and eosin; FCRwe, whole-enterprise FCR; GWP, global warming potential; EP, eutrophication potential; AP, acidification potential; LO, land occupation; CE, circular economy; LCA, life cycle assessment; LC-MS/MS, liquid chromatography–tandem mass spectrometry.

**Table 5:** Integration of LCA environmental impact categories with FCR-mediated mycotoxin effects and expected MMDA benefits in pig production.

LCA Impact Category	Primary Driver in Feed	Effect of Mycotoxicosis (↑ FCR)	FCR–Impact Correlation	Expected MMDA Benefit (↓ FCR)
GWP (kg CO_2_-eq)	N_2_O from N fertilizers; CO_2_ from fuel	↑ feed demand → ↑ GHG per kg pig	>0.95 [6,7]	~5% GWP reduction per 0.2 unit FCR improvement [5,7]
EP (kg PO_4_-eq)	N & P in excess feed → manure	↑ feed → ↑ N & P excretion per kg gain	>0.95 [6,7]	Proportional ↓ N & P excretion
AP (kg SO_2_-eq)	NH_3_ from manure; SO_2_ from fuel	↑ manure volume per kg gain	>0.95 [6,7]	↓ manure volume per kg gain
Land occupation (m^2^·yr)	Crop area for feed production	↑ feed required → ↑ land use per kg pig	>0.90 [6]	↓ crop area required per kg pig
Food safety/carry-over	Mycotoxin residues in pork (OTA, AFB1, FB1)	↑ tissue residues → ↑ consumer risk and condemnations	N/A (direct pathway)	↓ residues → ↓ food safety risk and slaughter waste
CE feed enabling	Safe use of co-products and contaminated fractions	Without MMDA: CE by-products cannot be safely used	N/A (enabling function)	With MMDA: circular feed formulations viable

MMDA, multicomponent mycotoxin-detoxifying agent; FCR, feed conversion ratio; GWP, global warming potential; EP, eutrophication potential; AP, acidification potential; N_2_O, nitrous oxide; NH_3_, ammonia; OTA, ochratoxin A; AFB1, aflatoxin B1; FB1, fumonisin B1; CE, circular economy; N/A, not applicable.

**Table 6:** Alignment of anti-mycotoxin agents, circular economy (CE) feed strategies, and life cycle assessment (LCA) outcomes in pig production with the UN Sustainable Development Goals (SDGs). MMDA, multicomponent mycotoxin-detoxifying agent; FCR, feed conversion ratio; GWP, global warming potential; EP, eutrophication potential; LO, land occupation; IARC, International Agency for Research on Cancer; LC-MS/MS, liquid chromatography–tandem mass spectrometry; TBARS, thiobarbituric acid reactive substances; CARBS, protein carbonyls; TAC, total antioxidant capacity.

SDG	Relevant Target	Mechanism in Pig Sector	Evidence/Metric	Key References
SDG 2: Zero Hunger	Target 2.1 (food access); 2.4 (sustainable food production)	MMDA restores FCR, protecting feed-to-protein conversion efficiency; CE by-product valorization reduces feed cost 10–25%	FCR improvement documented in field piglet trials; safe DDGS/former foodstuff inclusion enabled by MMDA	[2,13,28]
SDG 3: Good Health and Well-Being	Target 3.d (food safety and health security)	Reduction in AFB1, OTA, FB1 residues in pork; protection of consumer from IARC-classified mycotoxin carcinogens	LC-MS/MS slaughter analysis: FB1 2.91–8.30 μg/kg, OTA 0.54 μg/kg in commercial finisher pigs	[14,26]
SDG 6: Clean Water	Target 6.3 (water quality); 6.6 (aquatic ecosystems)	FCR improvement reduces N & P excretion per kg pig produced → lower eutrophication potential (EP)	FCR–EP correlation >0.95 across 74 LCA studies; proportional N/P reduction with each FCR unit improvement	[6,41]
SDG 8: Decent Work and Economic Growth	Target 8.2 (productivity and innovation); 8.3 (agricultural value chains)	Mycotoxin management protects farm income; CE feed strategies enable bio-based industry growth (USD 7.7 trillion by 2030)	Mycotoxin losses USD 1.4 billion/yr (USA); reduction in condemnations, veterinary costs, reproductive failures	[2,28]
SDG 12: Responsible Consumption and Production	Target 12.3 (food waste halving); 12.4 (chemical management)	MMDA enables safe use of DDGS, brewers’ grains, former foodstuffs; prevents DON-induced feed refusal/emesis (10–30% intake loss)	DON in 72% of DDGS samples (mean 1.84 mg/kg); MMDA prevents feed waste and closes biomass loops	[10,11]
SDG 13: Climate Action	Target 13.2 (integrate climate measures into policy)	FCR restoration by MMDA reduces GHG per kg pork; CE ingredient substitution cuts fertilizer-derived N_2_O and land-use emissions	Feed = 22–95% of pork GWP; FCR–GWP correlation >0.95; dairy probiotic LCA benchmark: −2.9% carbon footprint	[6,7,33]
SDG 15: Life on Land	Target 15.1 (sustainable ecosystems); 15.2 (halt deforestation)	CE feed strategies reduce land occupation (LO) per kg pork; fewer virgin cereal inputs → less pressure on natural habitats and soy-linked deforestation	FCR–LO correlation >0.90; 5–10% LO reduction achievable via FCR improvement + CE ingredient substitution	[6,40]
SDG 17: Partnerships for the Goals (One Health)	Target 17.14 (policy coherence for sustainable development)	One Health framework (FAO/UNEP/WHO/WOAH Joint Plan of Action) integrates animal, human and ecosystem health; mycotoxin management is priority action area	Field biomarker evidence (TBARS, CARBS, TAC) across sows and piglets provides cross-species One Health metrics	[5,12,13]

## Data Availability

No new data were created or analyzed in this study. Data sharing is not applicable to this article.
